# Choledocholithiasis Following an Orthotopic Liver Transplant: A Case Report and Brief Review of the Literature

**DOI:** 10.7759/cureus.86915

**Published:** 2025-06-28

**Authors:** Imran Khokhar, Eldia Delia, Gisha Mohan, Anish Paudel, Saraswathi Lakkasani

**Affiliations:** 1 Internal Medicine, Tower Health - Reading Hospital, West Reading, USA; 2 Internal Medicine, Suburban Community Hospital, Norristown, USA; 3 Gastroenterology, Saint Michael's Medical Center, Newark, USA

**Keywords:** cbd stones, cbd strictures, ercp, liver transplantation, orthotopic liver transplant complications

## Abstract

With advancements in transplant surgery, an orthotopic liver transplant remains the definitive treatment for end-stage liver disease. However, biliary complications, particularly strictures and choledocholithiasis, are among the most common causes of post-transplant morbidity and can significantly impact graft survival. Prompt recognition and timely intervention are essential to prevent progression to graft failure or the need for re-transplantation. We report a case of an orthotopic liver transplant patient who developed choledocholithiasis secondary to a duct-to-duct anastomotic biliary stricture. The patient was successfully managed with endoscopic retrograde cholangiopancreatography and biliary duct stent placement. This case highlights the importance of clinician awareness and the role of endoscopic therapy in the effective management of post-transplant biliary complications.

## Introduction

Orthotopic liver transplantation (OLT) is the gold standard for treating end-stage liver disease, with improvements in surgical techniques, immunosuppressive therapy, and postoperative care, significantly enhancing survival outcomes over time [1,2]. Despite these advances, biliary complications remain the most common cause of post-transplant morbidity and mortality [3,4]. Among these, biliary strictures and choledocholithiasis are particularly prevalent, often presenting with nonspecific symptoms or abnormal liver function tests [5,6].

These complications can arise due to ischemia, anastomotic technique, and immunosuppressant effects, or bile stasis, and are more frequently seen in duct-to-duct anastomoses [3,7]. The development of biliary stones is commonly secondary to strictures and may lead to significant consequences, including cholangitis, biliary obstruction, and even graft failure if not promptly managed [5,8]. Imaging modalities such as magnetic resonance cholangiopancreatography (MRCP) and computed tomography play key roles in diagnosis, while endoscopic retrograde cholangiopancreatography (ERCP) and endoscopic ultrasound remain the cornerstone for both diagnostic evaluation and therapeutic intervention [9].

## Case presentation

A 55-year-old woman with a past medical history of nonalcoholic steatohepatitis (NASH) cirrhosis, status post orthotopic liver transplant in 2013, chronic kidney disease (CKD) stage III, type 2 diabetes mellitus, and obstructive sleep apnea and no history of alcohol use disorder, presented to the emergency department (ED) with fever and pain in the right posterior chest wall. According to the patient, the fever and right chest wall pain progressively got worse over the last week prior. 

On admission, vital signs were as follows: temperature, 102.9°F; blood pressure, 145/67 mmHg; pulse, 95 beats per minute; respiratory rate, 16 breaths per minute; and SpO2, 98% on room air. A pertinent physical exam revealed a chevron incision on the abdominal wall and mild tenderness in the right hypochondrium and right posterior chest wall.

Significant lab values were notable for hemoglobin 10.4 gm/dL (reference: 13.5-17.5 gm/dL), hematocrit 23.2% (reference: 41-53%), creatinine 1.65 mg/dL (baseline ~1.0 mg/dL; reference: 0.7-1.3 mg/dL), alkaline phosphatase 621 U/L (reference: 44-147 U/L), alanine aminotransferase (ALT) 92 U/L (reference: 7-56 U/L), aspartate aminotransferase (AST) 81 U/L (reference: 10-40 U/L), gamma-glutamyl transferase (GGT) 636 U/L (reference: 9-48 U/L), total bilirubin 10.7 mg/dL (reference: 0.1-1.2 mg/dL), albumin 3.7 g/dL (reference: 3.5-5.0 g/dL), total protein 6.8 g/dL (reference: 6.0-8.3 g/dL), cholesterol 158 mg/dL (reference: <200 mg/dL), triglycerides 142 mg/dL (reference: <150 mg/dL), high-density lipoprotein (HDL) 49 mg/dL reference: >40 mg/dL for men), and low-density lipoprotein (LDL) 81 mg/dL (reference: <100 mg/dL for men).

**Figure 1:**
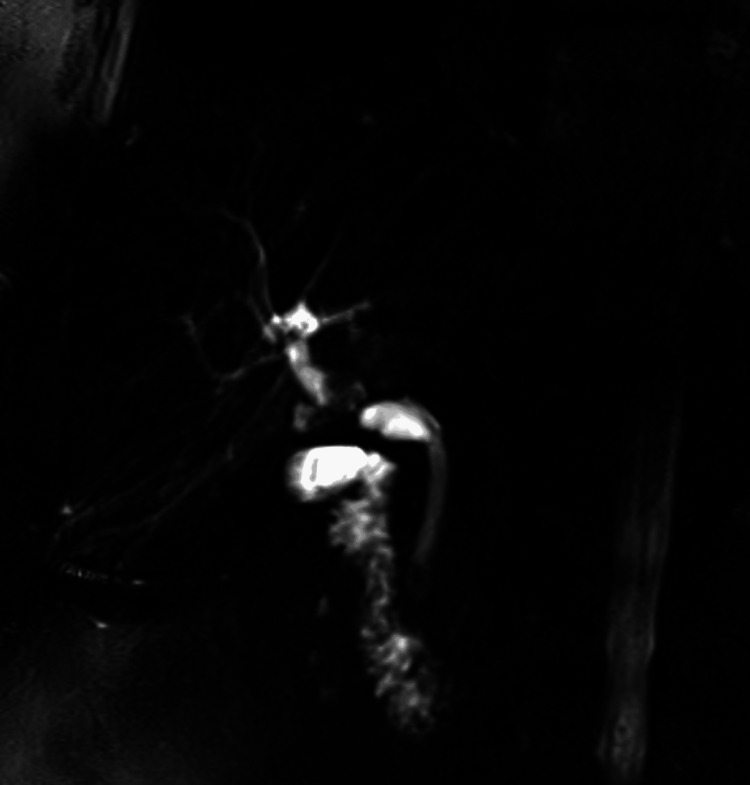
Magnetic resonance cholangiopancreatography (MRCP) showing duct-to-duct stricture and dilatation of the cystic duct stump

The patient was admitted for further evaluation, and the transplant team was consulted for any specific recommendations. The obstructive cholestatic pattern, indicated by elevated ALP and GGT, warranted further workup with liver ultrasound (US) and MRCP. Liver US showed hepatic steatosis and patent vasculature. MRCP revealed 6 mm and 4 mm stones in the biliary confluence with dilated cystic duct stump, and another 5 mm stone in the distal common bile duct (Figure 1).

**Figure 2:**
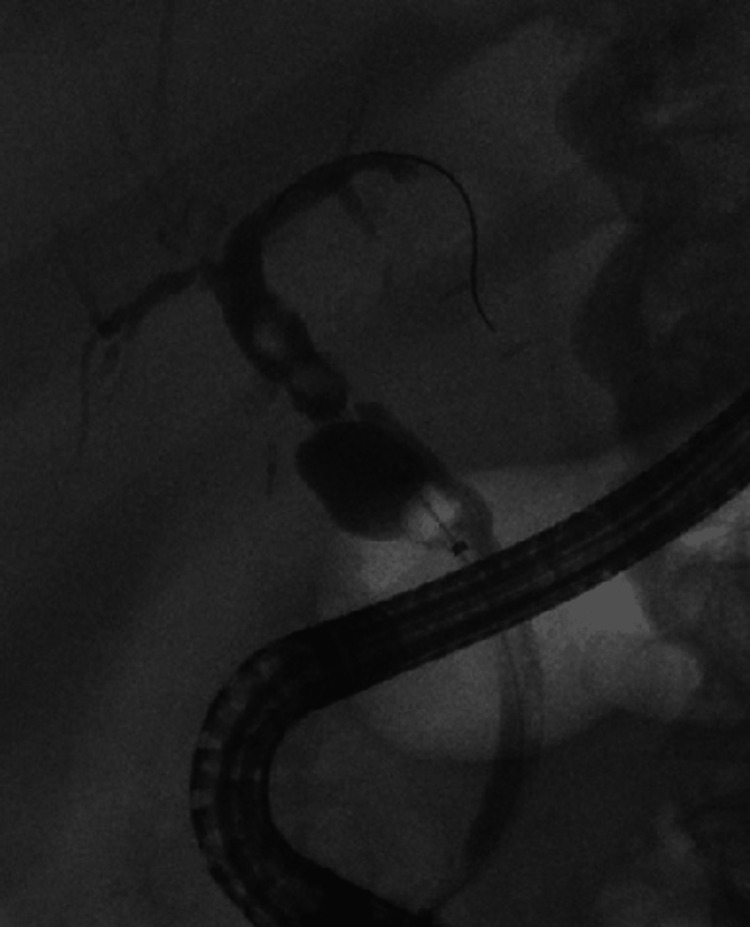
Endoscopic retrograde cholangiopancreatography (ERCP) showing filling defects in proximal and distal common bile duct (CBD) along with strictures in duct-to-duct anastomosis and dilatation of cystic stump

A gastroenterology and hepatology team was consulted and recommended ERCP. During ERCP, biliary cannulation was achieved by sphincterotomy and papillary balloon dilation. A single severe localized biliary stricture (leading to obstructive jaundice with common bile duct (CBD) stone) was found at the duct-to-duct post-liver transplant anastomosis (Figure 2).

**Figure 3:**
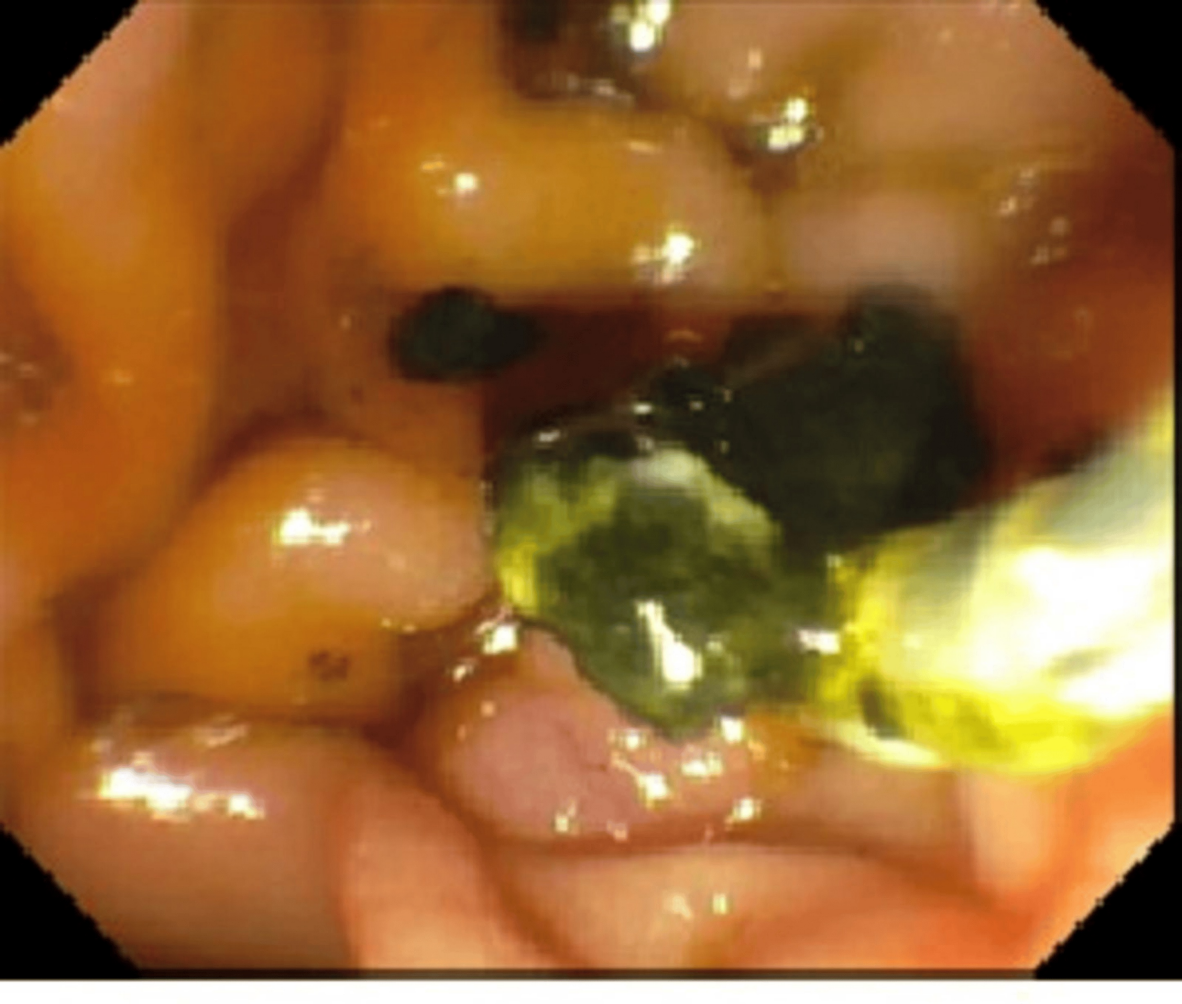
Endoscopic retrograde cholangiopancreatography (ERCP) showing extraction of stones from the common bile duct (CBD) after sphincterotomy

There were filling defects consistent with stones both proximal to the anastomosis as well as in the distal CBD. A 4 mm and 6 mm dilation was performed for the removal of three stones (Figures 2, 3).

**Figure 4:**
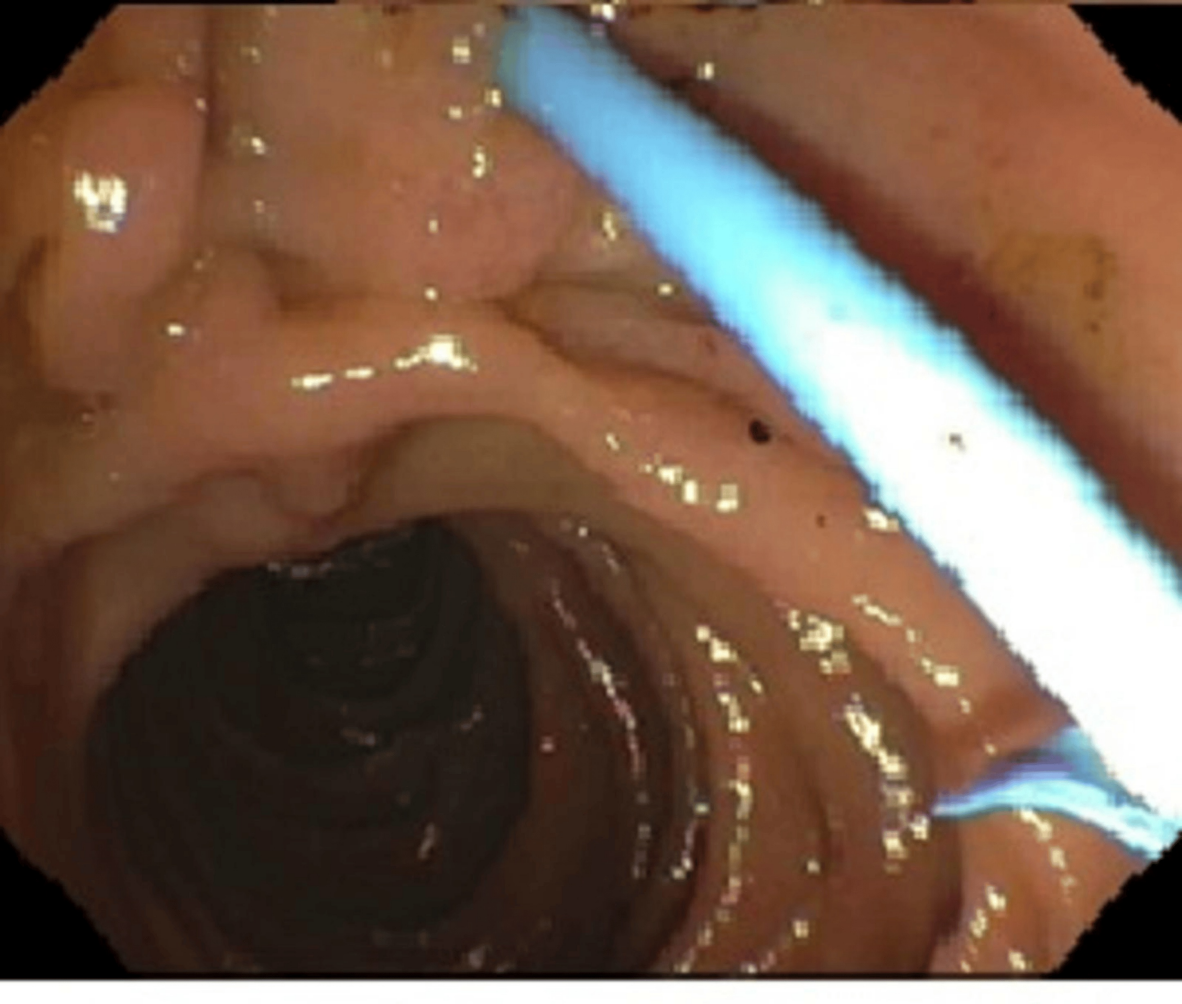
External flap plastic stent in the common bile duct after dilation and removal of stones in the biliary duct

A brushing biopsy was taken to rule out malignant stricture. One transpapillary 7 Fr x 15 cm plastic stent with a single external flap and a single internal flap was placed into the left hepatic duct (Figure 4). 

The procedure was uneventful, and the patient experienced an uncomplicated recovery. She was asked to return for follow-up in the next 8-10 weeks for repeat anastomotic stricture dilation, removal of any remnants of stone, and possible stent exchange.

## Discussion

A liver transplant is the gold standard treatment for end-stage liver disease, NASH, and acute liver failure. The most common technique utilized is OLT, in which the native diseased liver is replaced by a healthy donor liver [1]. Despite improvements in surgical techniques and immunosuppressants, there are various complications associated with OLT. Those most commonly seen are vascular, biliary, ischemic, and infectious in origin. Several uncommon complications include fluid collection, hematoma formation, lymphoproliferative disorders, and recurrent tumors. Biliary complications remain among the most common adverse outcomes following OLT. Any biliary complication is reported in approximately 10-25% of transplant recipients, though some studies cite a range of 5-20%. Anastomotic strictures occur in approximately 4-13% of recipients of deceased donor liver transplants and 18-40% of living donor liver transplants. Non-anastomotic strictures, often related to ischemia or immunologic injury, occur in 0.5-10% of patients, with some reports documenting incidences as high as 20%. Biliary stones or sludge are noted in approximately 2-18% of liver transplant recipients [2].

Biliary complications include biliary stricture, stones, leakage, and obstruction. Initial signs of biliary complications in asymptomatic liver transplant patients include elevated serum aminotransferases, bilirubin, ALP, and GGT levels. Physical examination may reveal right upper quadrant tenderness and skin excoriation due to pruritus, jaundice, and ascites. However, tenderness may not be present in some patients due to hepatic denervation and immunosuppression [3].

During the early postoperative period, bile leak and ischemia are more common, whereas the late postoperative period tends to give rise to biliary stricture complications [4]. At an incidence of 1.8-18%, bile duct stones are the third most common biliary complication to occur after OLT and are associated with significant morbidity [5,6]. If not identified and treated promptly, they can lead to hepatobiliary tract obstruction and infection, causing graft failure and increasing the re-transplantation rate [7,8].

There are different causes of CBD stone formation in the literature. In our patient, the identified cause was biliary duct stricture. Previous case-control studies have found that CBD stones can be due to biliary duct strictures formed by surgical or mechanical factors [6,7]. The other causes include decreased bile acid synthesis due to immunosuppressive agents (e.g., cyclosporine) promotes stone formation [7], bile duct stricture and stone formation due to supersaturation of bile from cholesterol ≥ 200 mg/dL and triglyceride ≥ 150 mg/dL [7], and hepatic artery thrombosis from warm and cold ischemia [8,9].

A variety of diagnostic and therapeutic modalities are available for managing patients with choledocholithiasis. As new technology is discovered, capabilities expand, and adverse effects decrease. MRCP is considered the gold standard for diagnosing biliary complications in these patients, with a sensitivity of 96% and specificity of 94% [10]. ERCP is a useful diagnostic and therapeutic modality for choledocholithiasis, though it may cause acute pancreatitis or hemobilia [5]. Single-operator cholangioscopy (SpyGlass 2) with ERCP has been used more recently, though its use is limited in the treatment of intrahepatic bile duct stones [11]. Other treatments include interventional percutaneous radiologic procedures and open surgery [10-15]. Percutaneous transhepatic cholangioscopy/choledochoscopy in combination with laser/mechanical lithotripsy and basket stone retrieval are found to be very effective with fewer adverse effects [7,10]. 

## Conclusions

In orthotopic liver transplant patients, biliary complications such as CBD stone formation are associated with a high risk of morbidity and mortality. Early diagnosis and treatment are crucial to prevent adverse events. A detailed history and examination and correlation of labs and imaging are important to make the correct diagnosis. Appropriate surgical techniques are the best preventive measures to decrease duct-to-duct anastomotic strictures and stone formation. Clinicians should be aware of these complications to minimize the risk of graft failure and select the most appropriate treatment modality for each patient.
